# Effective Statistics Are Essential for Strengthening a Paper in Brain and Behavior

**DOI:** 10.1002/brb3.70727

**Published:** 2025-09-09

**Authors:** Michal Ordak, Raffaella Bosurgi

**Affiliations:** ^1^ Department of Pharmacotherapy and Pharmaceutical Care, Faculty of Pharmacy Medical University of Warsaw Warsaw Poland; ^2^ John Wiley & Sons Ltd Bognor Regis UK

**Keywords:** behavior, brain, statistical recommendations

Brain and Behavior, launched in 2011 by Andrei V. Alexandrov, is an open‐access, multidisciplinary journal dedicated to the rapid publication of original research in neurology, neuroscience, psychology, and psychiatry. Since then, the journal has expanded its scope and today it publishes studies on brain function and behavior in relation to health, social, political systems, and the environment. It accepts research in clinical and basic sciences, including descriptive studies, preliminary research, and work that involves reproducibility. The goal is to support authors in publishing research while meeting high‐publication standards. Brain and Behavior's philosophy is to disseminate good and sound science. A study to be good doesn't have to be novel or ground breaking but it has to be robust and has to follow rigour in methodology and statistics. Demonstration that a result can be replicated in independent studies is key in validating novel studies and good statistics can enhance the overall impact of the studies (Alexandrov 2011). Statistics plays three important roles in brain studies. They include the study of differences between brains in distinctive populations, the study of variability in the structure and functioning of the brain, and the study of data reduction on large‐scale brain data. These areas are illustrated through research in brain connectivity, information flow, large‐scale neuroimaging data, and predictive modeling. After outlining these roles, one can consider how statistical science continues to support brain decoding and how collaboration between statistical and neurobiological communities may contribute to addressing future questions (Chén 2019). The most recent data suggest that we are dealing with low quality in statistical reporting (Ordak 2024). By providing a set of basic statistical recommendations we aim to guide authors to help improve the quality and transparency of future submissions to Brain and Behavior.

One key recommendation concerns effect size measures. A *p* value indicates whether an observed effect is unlikely due to chance, but it does not communicate the strength or importance of the effect. Effect sizes allow researchers to determine how large a difference or association is and whether it may be practically or clinically meaningful. Including them supports interpretability and enables comparisons between studies. An example can be found in a study published in Brain and Behavior by Elkjaery et al., where the authors tested the emotional impact of sequentially induced anger and anxiety—emotions associated with opposing action tendencies (approach vs. avoidance) using independent samples *t*‐tests and mixed‐effects ANOVAs. Emotional impact in this context refers to changes in emotional states (e.g., anger, anxiety, and irritability) and behavioral inclinations, assessed through visual and verbal measures of action tendencies, following experimental manipulations designed to test the incompatible response hypothesis. They examined associations between outcome measures through correlation analysis and reported effect sizes using Pearson's *r*, Cohen's *d*, and partial eta squared. They interpreted these values based on the thresholds proposed by Cohen for small, medium, and large effects (Elkjaer et al. 2023). As Jacob Cohen emphasized, “The primary product of a research inquiry is one or more measures of effect size, not *p* values” (Cohen 1990). This is because effect size measures provide information about the magnitude and practical significance of a finding, helping readers to understand whether a result is not only statistically significant but also meaningful in real‐world or clinical contexts. In contrast, *p* values alone do not convey how strong or relevant an effect is.

Another recommendation concerns the choice of appropriate post‐hoc tests in the context of multiple comparisons. Selecting the correct post‐hoc procedure depends on assumptions such as distribution type and homogeneity of variance. A common mistake is applying a post‐hoc test without verifying these assumptions, which can lead to significantly different outcomes. A well‐executed example can be found in a study published in Nature Communications on developmental mechanisms in the embryonic brain. For comparisons involving parametric data, they used one‐way ANOVA followed by Tukey's test. For nonparametric data, they applied the Kruskal–Wallis test followed by Dunn's test. They clearly stated which tests were used under which conditions, and their rationale reflected statistical appropriateness, such as variance homogeneity (László et al. 2019).

Testing and reporting statistical assumptions is also an important aspect to consider. These include, for example, assumptions such as normality, sphericity, homogeneity of variance, equal group sizes, and the absence of multicollinearity among predictors. One of the most frequent mistakes is using parametric tests even when the assumptions are visibly violated. It is therefore recommended not only to state which tests were used, but also to explain how their assumptions were evaluated and whether they were met. A good example of such proper reporting appears in a study by Grisoni et al. in Journal of Neuroscience, examining semantic processing during language comprehension and production. The authors reported key information for ANOVA analyses, including *F*‐values, degrees of freedom, *p* values, and effect sizes. They checked for violations of sphericity, and when necessary, applied the Greenhouse–Geisser correction, explicitly reporting the correction values (Grisoni et al. 2024). This approach offers clarity about how assumption violations were addressed.

Statistical outcomes can be influenced by the role of outliers which can change effect estimates, or distort conclusions. This is particularly relevant in correlation analyses. In such contexts, a single data point that deviates from the general distribution can inflate the correlation coefficient, create an artificial sense of association, or even reverse the direction of the correlation—from negative to positive or vice versa. For this reason, authors should analyze the influence of outliers and report how they were handled during statistical analysis (Makin and Orban de Xivry 2019). Do we have a good example in Brain and Behavior of how the role of outliers?

It is worth to mention also some aspects related to meta‐analysis. For instance, it is essential to remember that the *I*‐squared value is not an absolute measure of heterogeneity. Instead, it quantifies the proportion of total variability that is due to heterogeneity between studies rather than random error. Furthermore, one more recommendation concerns the analysis of interaction effects, such as those encountered in ANOVA or regression. It is not sufficient to simply state that an interaction was tested. Authors should explain how the interaction was analyzed and interpreted, including whether any follow‐up tests or decompositions were carried out. It is also advisable to pay attention to the choice of descriptive statistics. In nonparametric contexts, it is not appropriate to report only means and standard deviations. Instead, authors should use statistics such as the median and interquartile range to more accurately reflect the distributional characteristics of the data.

Handling of missing data deserves also a particular attention. As a matter of fact, it should be clearly described, including whether observations were excluded, imputed, or handled using specific statistical models. The chosen method should be explained so that readers can properly assess its influence on the results. Clear reporting of statistical tests is also crucial. Authors should specify which test was used and where it was applied. For example, a notation like “*U* = 12; *p* = 0.03” enables readers to understand precisely which test was conducted and to evaluate the appropriateness and strength of the result. Clarifying the purpose of each analysis—what specific hypothesis or research question each test was meant to address, is crucial. Unfortunately, it is still common to see lists of tests without any explanation of their role in the analysis.

Last but not least, when using advanced statistical methods, authors should briefly explain why these approaches were selected and what they involve in general terms. This will allow readers to follow the logic of the analysis, which is particularly important given that even basic statistical concepts are sometimes poorly understood. For advanced methods, such explanation becomes even more critical (Ordak et al. 2024; Ordak 2022).

We hope that these basic statistical recommendations will guide authors in preparing their papers and to help them focusing on key statistical considerations before submitting their work to Brain and Behavior.

## Author Contributions

**Michal Ordak**: conceptualization, methodology, writing – review and editing, writing – original draft, visualization. **Raffaella Bosurgi**: writing – review and editing, conceptualization.

## Conflicts of Interest

The authors declare no conflicts of interest.

## Peer Review

The peer review history for this article is available at https://publons.com/publon/10.1002/brb3.70727

## Data Availability

Data sharing is not applicable to this article as no new data were created or analyzed in this study.
